# EMG-Driven Musculoskeletal Modelling Framework for Virtual Simulation of Upper Limb Activation-Modulated Impairment Scenarios

**DOI:** 10.3390/medicina62030530

**Published:** 2026-03-12

**Authors:** Dovydas Cicėnas, Kristina Daunoravičienė

**Affiliations:** Department of Biomechanical Engineering, Faculty of Mechanics, Vilnius Gediminas Technical University, Plytinės g. 25, LT-10105 Vilnius, Lithuania

**Keywords:** EMG-driven musculoskeletal modelling, movement quality assessment, neuromuscular impairment, upper limb biomechanics, rehabilitation engineering

## Abstract

*Background and Objectives:* Surface electromyography (EMG) is widely used to assess muscle activation. However, direct interpretation of its functional biomechanical consequences remains challenging. This study aimed to develop and evaluate an EMG-driven musculoskeletal simulation framework for investigating how controlled modifications of muscle activation patterns influence joint-level biomechanics in the upper limb. The objective was not to reproduce specific clinical pathologies but to enable systematic virtual scenario analysis of activation-dependent movement alterations. *Materials and Methods:* Surface EMG signals were recorded from five healthy adults (3 males, 2 females; age 22 ± 1 years) during cyclic elbow flexion/extension tasks using a wireless system (sampling frequency: 2000 Hz). Processed and normalized EMG envelopes were directly applied as prescribed neural inputs in forward dynamic simulations implemented in OpenSim, without optimization-based muscle recruitment. Controlled virtual scenarios were generated through parametric modification of activation signals to represent reduced activation capacity, increased antagonist co-activation, spasticity-like activation modulation, and tremor-like oscillatory modulation. Joint kinematics, joint moments, and movement stability were evaluated. A Movement Quality Index (MQI) was introduced as a comparative research metric integrating biomechanical performance indicators. Simulations were deterministic and analyzed descriptively. *Results:* Distinct activation modifications produced characteristic kinematic and kinetic responses. Reduced activation capacity decreased simulated joint moment output, increased co-activation altered joint moment timing and mechanical stability, and tremor-like oscillatory modulation generated periodic fluctuations in joint kinematics and kinetics. The MQI enabled quantitative differentiation between simulated scenarios and severity levels within the controlled modelling framework. *Conclusions:* The proposed EMG-driven forward dynamic simulation framework provides a methodological platform for controlled virtual scenario analysis of activation-dependent biomechanical changes. The findings highlight the sensitivity of joint-level mechanics to altered muscle activation patterns, within the deterministic modelling environment. The framework is intended for research-oriented biomechanical investigation and hypothesis testing rather than direct clinical diagnosis of neuromuscular disorders.

## 1. Introduction

Accurate assessment of muscle activation during movement is important for understanding motor control and supporting rehabilitation research strategies. Surface electromyography (EMG) provides quantitative information about muscle activation patterns during functional tasks. However, translating EMG signals into joint-level biomechanical interpretation remains challenging.

Although EMG is widely used in both research and clinical settings, the relationship between recorded activation patterns and resulting joint mechanics cannot be directly inferred from signal amplitude alone. Computational musculoskeletal modelling offers a means of linking activation patterns to simulated muscle forces, joint moments, and kinematics within a controlled framework.

Despite extensive research on surface EMG, integration of experimentally recorded activation signals with subject-specific musculoskeletal simulations remains technically demanding and is not routinely implemented in applied movement analysis. Consequently, the biomechanical implications of altered activation patterns are often investigated separately from computational musculoskeletal modelling.

To summarize the main limitations of commonly used clinical assessment tools and illustrate the complementary roles of conventional assessment and modelling-based approaches, Table 1 provides a comparative overview of widely used methods in upper limb evaluation.

Conventional clinical EMG examinations, whether needle EMG or surface EMG, are primarily designed to detect denervation, myopathic changes or abnormal spontaneous activity, rather than to quantify muscle function during everyday movements. Standard reports focus on motor unit morphology and recruitment patterns in static or simple tasks, and they provide continuous, task-level metrics of muscle contribution during functional activities such as reaching or elbow flexion/extension [5].

From a signal perspective, EMG measurements are influenced by factors such as crosstalk, electrode placement variability, and skin-electrode impedance, which complicate interpretation of signal amplitude as a direct proxy for muscle force [6]. As emphasized in the literature, meaningful clinical use of EMG requires biomechanical context, including information on joint kinematics and loading conditions, to distinguish appropriate muscle activation from compensatory or pathological patterns [4,5].

These challenges are summarized in Table 2, which outlines key educational, technical, and methodological barriers limiting routine integration of surface EMG with biomechanical modelling.

These barriers reflect literature-reported limitations of EMG integration in routine clinical contexts and should not be interpreted as inherent shortcomings of the EMG methodology itself.

Moreover, although advanced EMG analyses (e.g., timing of synergies, co-activation indices, frequency-based markers of fatigue) are well established in the research literature, they are rarely implemented at the point of care. Toepp et al. note that many clinicians find EMG “interesting but not actionable” because raw waveforms do not directly translate into clear diagnostic or prognostic information [8]. Thus, despite delivering detailed electrophysiological information, current clinical EMG practice does not routinely provide a direct joint-level mechanical interpretation of muscle activation during functional tasks.

Dynamometry provides reliable estimates of maximal force capacity but does not capture how muscles are coordinated during functional, multi-joint movements. As a result, near-normal strength values may coexist with impaired movement quality driven by abnormal activation patterns or excessive co-contraction.

Similarly, goniometric assessment provides discrete measurements of joint range of motion but offers limited insight into movement dynamics or neuromuscular coordination [9].

This study aimed to develop and evaluate an EMG-driven musculoskeletal simulation framework that uses experimentally recorded surface EMG as direct control input for forward dynamic simulations. The framework enables systematic and controlled modification of muscle activation patterns to investigate their biomechanical consequences at the joint level. A Movement Quality Index (MQI) was introduced as a comparative research metric to quantitatively assess differences between simulated activation scenarios.

The proposed approach is intended as a methodological platform for controlled virtual scenario analysis rather than as a direct clinical diagnostic tool.

## 2. Methods

### 2.1. Overview of an EMG-Driven Musculoskeletal Modelling Workflow

**Figure 1:**
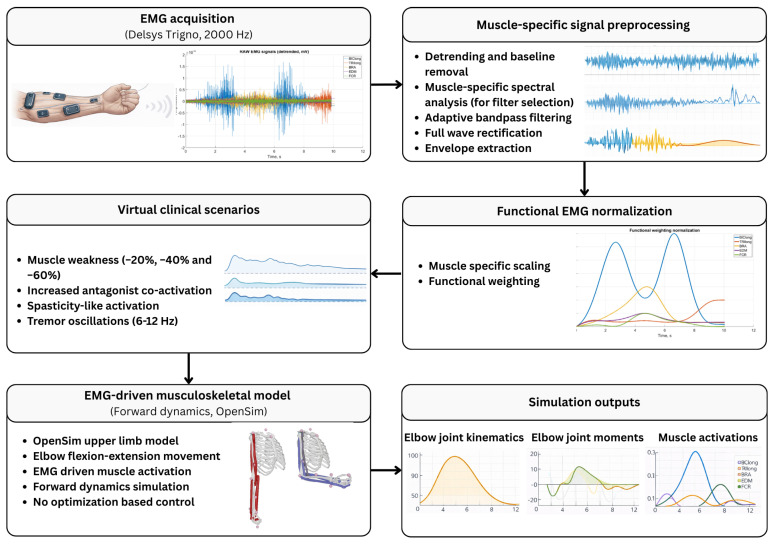
Workflow of the proposed EMG-driven virtual clinical simulation framework for neuromuscular impairment analysis.

The proposed EMG-driven musculoskeletal modelling workflow enables controlled simulation and functional analysis of neuromuscular impairment mechanisms based on experimentally recorded muscle activation patterns. The workflow consists of four stages: (i) experimental data acquisition and EMG pre-processing, (ii) EMG-driven model implementation, (iii) simulation of virtual neuromuscular impairment scenarios, and (iv) biomechanical outcome assessment (Figure 1). EMG signals were pre-processed using standard procedures to obtain muscle activation profiles suitable for musculoskeletal simulation. The processed EMG signals were treated as prescribed activation inputs and were not adjusted or optimised to match kinematic or kinetic targets. In the second stage, an upper limb musculoskeletal model was implemented in the OpenSim (version 4.5, Stanford University, Stanford, CA, USA) environment. EMG-derived muscle activations were mapped directly to the corresponding model actuators and used as the sole control inputs for forward dynamics simulations. No optimization-based control strategies were applied.

Muscles without available EMG recordings were not actively driven (activation set to zero) and contributed only through passive muscle–tendon properties and passive joint mechanics. This assumption represents a modelling simplification adopted to isolate the mechanical effects of experimentally driven activation patterns under controlled simulation conditions. Low-level tonic activation and residual co-activation may occur in vivo, and that assigning zero activation to non-instrumented muscles may lead to underestimation of baseline joint stiffness and stabilization effects. However, this approach was selected to preserve model transparency and interpretability within the scope of the present methodological study.

In the third stage, virtual neuromuscular impairment scenarios were generated by systematic and physiologically motivated modifications of the EMG activation patterns. These scenarios were designed to represent distinct impairment mechanisms, including reduced agonist activation (muscle weakness), increased antagonist co-activation, spasticity-like activation patterns, and tremor-like oscillatory activity.

All simulations were performed using the same model structure and task conditions to ensure that observed differences in biomechanical outcomes arose exclusively from changes in neuromuscular activation. In the final stage, the biomechanical consequences of each simulation were evaluated using joint kinematics, total joint moments, and a Movement Quality Index (MQI). This integrated assessment enabled systematic and quantitative comparison of how different neuromuscular impairment mechanisms affect joint-level mechanics and movement quality under otherwise identical conditions.

### 2.2. Experimental Data Acquisition and EMG Pre-Processing

Surface electromyography (EMG) signals were acquired from selected upper limb muscles during cyclic elbow flexion–extension tasks performed by five healthy adult (3 males, 2 females; age 22 ± 1 years) participants. Surface EMG signals were recorded using a Delsys Trigno wireless system (Delsys Inc., Natick, MA, USA) at a sampling rate of 2000 Hz.

Participants performed repeated elbow flexion–extension movements at a self-selected comfortable pace without external loading while seated, with the upper arm supported to minimize shoulder motion. Surface EMG was recorded using bipolar electrodes placed according to standard anatomical guidelines after skin preparation to reduce impedance [10].

All preprocessing steps followed commonly accepted procedures in EMG signal processing to ensure physiological plausibility of the resulting activation signals. To enable direct use of EMG signals as control inputs for the musculoskeletal model, the processed EMG envelopes were normalized and time-aligned with the simulated movement cycles. Importantly, the resulting activation profiles were treated as prescribed inputs and were not modified, scaled, or optimized to fit kinematic or kinetic targets. The pre-processed EMG activation signals served as the sole neural drive for the corresponding muscles in the EMG-driven musculoskeletal simulations.

Raw EMG signals were band-pass filtered using a zero-lag, fourth-order Butterworth filter with cut-off frequencies of 20–450 Hz to remove motion artefacts and high-frequency noise while preserving physiologically relevant signal content. Following band-pass filtering, EMG signals were full-wave rectified and low-pass filtered using a zero-lag Butterworth filter (cut-off frequency: 5 Hz) to obtain linear envelopes representing muscle activation profiles. For tremor-related simulations, oscillatory components were superimposed on the normalized EMG envelopes after envelope extraction, rather than preserved through low-pass filtering. The choice of low-pass cut-off frequency was selected to preserve task-related activation patterns while avoiding excessive smoothing that could suppress oscillatory components relevant for tremor-related simulations.

Because linear envelope extraction involved low-pass filtering at 5 Hz, physiological tremor-frequency components were not preserved in the processed EMG signals. Instead, tremor-like oscillatory activation patterns were introduced parametrically after envelope extraction to enable controlled investigation of oscillatory activation effects rather than reconstruction of neurophysiological tremor mechanisms.

To enable inter-muscle and inter-condition comparison, EMG envelopes were normalized to the maximum activation observed for each muscle across all recorded trials. This normalization approach was selected to preserve relative activation timing and modulation patterns without imposing task-specific maximal voluntary contraction (MVC) assumptions, which are not always feasible or reliable in clinical populations. All EMG signals were temporally aligned to the corresponding movement cycles and resampled to match the simulation time step. No amplitude scaling, gain optimization, or feedback-based adjustment was applied after normalization.

This normalization strategy represents a deliberate trade-off, favouring within-subject and within-muscle comparative analyses of activation patterns over absolute force estimation or inter-subject comparability. The observed maximum activation does not necessarily reflect true neuromuscular capacity and may introduce scaling bias. This approach was selected to maintain internal consistency within controlled simulation scenarios.

### 2.3. Musculoskeletal Model and EMG-Driven Control Implementation

In the proposed framework, joint kinematics were not externally prescribed but emerged from forward dynamic simulation driven exclusively by EMG-derived activation inputs and musculoskeletal model dynamics. External kinematic data were used for validation purposes only and were not required for simulation control.

The simulation workflow consisted of: (1) EMG acquisition parameters and electrode placement; (2) band-pass filtering, rectification, and linear envelope extraction; (3) normalization procedures; and (4) generation of activation time-series inputs used to drive the musculoskeletal model.

A modified upper limb musculoskeletal model based on Holzbaur et al. was implemented in OpenSim and adapted for elbow flexion/extension analysis [11]. The model represents the upper limb using four primary elbow muscles: (1) biceps brachii (BIClong), (2) triceps brachii (TRIlong), (3) brachialis (BRA), (4) brachioradialis (BRD)—selected as the main contributors to elbow torque generation. Muscle activations were applied directly as OpenSim inputs using the corresponding actuator names. EMG-derived activations were prescribed as time-varying inputs, and no feedback, tracking, or error-minimization control was employed.

Muscle–tendon units spanning the elbow joint were represented using Hill-type muscle models with activation and contraction dynamics. No inverse dynamics based or optimization-based control strategies were applied. Muscle activations were not modified or scaled to reproduce experimental kinematics or joint moments. Muscles without EMG recordings were assigned zero neural activation and contributed only through passive muscle–tendon properties and passive joint mechanics. As a result, the simulated movement emerged solely from the interaction between experimentally derived muscle activation patterns, passive musculoskeletal properties, and task constraints.

### 2.4. Definition of Virtual Clinical Scenarios

To simulate activation-modulated virtual impairment scenarios, baseline normalized EMG signals were systematically modified based on literature-informed principles. All modifications were applied at the EMG signal level before musculoskeletal simulation, while the model structure and parameters remained unchanged. All scenarios represent virtual, isolated modifications of muscle activation patterns and do not aim to reproduce complete clinical pathologies.

#### 2.4.1. Muscle Weakness

To investigate the biomechanical consequences of reduced neuromuscular control, virtual reduced agonist activation (muscle weakness-like condition) scenarios were created by scaling normalized EMG activation signals of the elbow flexor muscles. Three levels of weakness were simulated, uniformly reducing agonist muscle activation by 20%, 40%, and 60% compared to the healthy baseline condition.

Previous EMG studies have shown that muscle weakness can be manifested by reduced EMG amplitude, with essentially preserved activation time, especially during submaximal voluntary tasks [12,13]. By modifying only EMG amplitude, the model allows us to isolate the mechanical consequences of reduced force generation without introducing additional coordination changes.

This representation should be interpreted as a first-order approximation of reduced effective moment-generating capacity rather than a full physiological reproduction of neurogenic or myogenic weakness mechanisms. The non-linear EMG-force relationship and potential compensatory activation strategies observed in clinical populations are acknowledged as limitations of this simplified modelling approach.

#### 2.4.2. Increased Antagonist Co-Activation

Co-activation scenarios were created by selectively increasing the EMG activation of antagonist muscles while maintaining the activation of agonist muscles at the same level as the baseline condition, in order to investigate the biomechanical effects of increased antagonist co-activation on movement. In this study, triceps brachii activation was increased by +20%, +40%, and +60% compared to the healthy baseline condition, while elbow flexor activation remained unchanged.

Increased EMG activity of antagonists during voluntary movement is a well documented phenomenon in neurological disorders and compensatory motor strategies, often interpreted as an attempt to increase joint stability at the expense of movement efficiency [14]. Surface EMG analyses consistently show increased indices of co-activation (co-contraction) in patients with impaired motor control, and therefore EMG-based enhancement of antagonist activation represents a modelling approximation consistent with reported EMG observations of increased co-activation in impaired motor control.

All simulations were performed using the same EMG-driven forward dynamics system, ensuring that the observed changes in joint mechanics were due solely to the altered activation of antagonist muscles.

#### 2.4.3. Spasticity-like Activation

Muscle spasticity was modelled by introducing an increased activation level together with reduced signal modulation. Spasticity-like behavior was simulated by introducing a tonic component of basal activation along with enhanced phasic activation pulses in selected muscles, resulting in EMG activation remaining elevated throughout the movement. This approach is consistent with EMG observations in individuals with upper motor neuron lesions, where spasticity is characterized by increased background muscle activity, enhanced reflex responses, and reduced modulation of activation during the movement cycle [15,16]. Although real spasticity involves complex reflex mechanisms that are dependent on movement velocity, activation-based EMG modulation is widely used in modelling studies as a first-order approximation to assess its mechanical consequences at the joint level.

#### 2.4.4. Tremor Simulation

The tremor-like activation pattern was introduced to simulate selected biomechanical features commonly associated with pathological tremor observed in neurological disorders, including involuntary rhythmic muscle activity and impaired coordination of agonists and antagonists during movement.

Oscillatory components were parametrically superimposed on normalized EMG envelopes after envelope extraction and normalization, prior to being used as control inputs for the model. Pathological tremor is characterized by rhythmic EMG pulses in specific frequency bands, which typically range from 4 to 12 Hz [17,18]. In the present study, these frequency characteristics were used as reference ranges for parametric modulation rather than direct physiological reconstruction. These parametrically defined oscillatory components were then applied to the processed activation signals used as control inputs for simulation. EMG-based tremor modelling has been widely described in both experimental and simulation studies, showing that oscillatory muscle activation translates into joint moment fluctuations and movement instability. The present implementation focuses on controlled biomechanical consequences of oscillatory activation rather than modelling the underlying neurophysiological origin of tremor.

These baseline activation profiles reflected physiologically-based voluntary control and were used as a reference (healthy) condition.

Tremor was applied to the biceps brachii, brachioradialis, and triceps brachii muscles. Involvement of both agonist and antagonist muscles was implemented to generate a tremor-like oscillatory interaction pattern, as tremor-like patterns in clinical conditions often manifests as oscillatory interactions between opposing muscle groups rather than isolated agonist activity. To further enhance pathological realism, the modulation of the antagonist muscle tremor was introduced with a 180° phase shift relative to the agonist component, thus promoting oscillatory compensation of joint moments and greater mechanical instability. This phase relationship was introduced to promote alternating agonist–antagonist moment fluctuations commonly observed in pathological tremor.

Three levels of tremor severity were simulated in this study, increasing the amplitude of the modulation relative to the baseline activation (20%, 40% and 60%).

Consistent with reported tremor phenomenology, the tremor-like modulation was not applied continuously throughout the simulation. Muscle tremor was simulated episodically to allow controlled analysis of tremor-induced mechanical instability during distinct movement phase. Tremor was activated in the middle flexion phase (20–60% of the movement cycle) and during the transition from maximum flexion to early extension (60–85% of the cycle). This method allowed for a systematic study of how pathological oscillatory muscle activity propagates through the neuromuscular system and affects joint kinematics and kinetics.

Each modified EMG dataset was saved as a separate control input file and used to drive forward dynamic simulations.

### 2.5. Simulation Output and Biomechanical Analysis

For each simulation, joint kinematics, prescribed muscle activation profiles, and net joint moments were extracted from OpenSim outputs. Elbow angle trajectories were time-normalized to the 100% movement cycle, defined from movement onset to completion of one flexion–extension cycle.

The following outcome measures were evaluated for all scenarios, with tremor-specific metrics assessed only in tremor-related simulations:

- Elbow range of motion (ROM);
- Peak elbow flexion angle and timing;
- Peak joint moment;
- Tremor amplitude and dominant frequency content of joint moment oscillations, where applicable.

All outcome measures were evaluated relative to the healthy baseline conditions to quantify impairment-induced deviations.

### 2.6. Elbow Joint Moment Estimation

Elbow joint moments were obtained from EMG-driven forward dynamics simulations performed in OpenSim software, without inverse dynamics or post hoc optimization. For each virtual clinical scenario (healthy, muscle weakness, increased co-activation, spasticity and tremor activation), the corresponding EMG-based control signals (in .sto format) were applied to a EMG-driven musculoskeletal model. Joint moments were calculated using OpenSim muscle analysis tools, extracting the elbow flexion/extension moment, which represents the resulting the net internal flexion–extension moment acting around the elbow joint, taking into account the combined effect of all agonist and antagonist muscle forces, as well as the passive joint contribution. Joint moment time series were exported from OpenSim and processed for analysis and visualization in MATLAB (R2024b, MathWorks, Natick, MA, USA).

### 2.7. Movement Quality Index

To provide an integrated measure of functional performance, a Movement Quality Index (MQI) was defined. The MQI was developed as a research-oriented comparative index integrating kinematic performance, joint moment generation, and movement stability into a unified metric for relative evaluation of simulated scenarios. Component metrics were selected based on their relevance to the specific impairment mechanism and were included only when applicable, with normalization ensuring comparability across scenarios. Each component metric was individually normalised to a unitless scale before aggregation to ensure comparability across biomechanical domains. Equal weighting was adopted to avoid introducing impairment-specific bias and to enable transparent comparison across different virtual scenarios.

Equal weighting of the kinematic, kinetic, and stability components was adopted to preserve methodological transparency and avoid impairment-specific bias. The MQI is not intended as a clinically validated outcome score but as an exploratory engineering metric for controlled comparative analysis. Future work may explore alternative weighting strategies or sensitivity analyses to tailor the index to specific functional contexts.

### 2.8. Statistical and Comparative Analysis

The simulation results were analyzed in a descriptive and comparative manner, evaluating different conditions. Inferential statistical testing was not applied because the study focuses on deterministic virtual simulations rather than repeated experimental observations. The reported results therefore represent comparative outcomes between controlled virtual scenarios rather than population-level estimates or probabilistic inferences. For each scenario, the outcome measures were compared to the healthy baseline condition which served as the reference condition for all relative comparisons. Correlation-based comparisons and deviation metrics were applied where appropriate to quantify differences in waveform shape relative to the reference trajectories.

All data processing, scenario generation, and post-simulation analysis were per-formed in MATLAB, while musculoskeletal simulations were executed in OpenSim.

## 3. Results

### 3.1. Virtual Muscle Weakness Scenario

EMG data were processed individually for each participant. For simulation analysis, a representative dataset was selected based on signal quality and movement consistency to ensure stable forward dynamic behaviour. The objective of the study was methodological scenario comparison rather than statistical generalization across subjects.

EMG signals were screened using predefined technical criteria, including stable baseline activity after detrending, absence of signal saturation or excessive motion artefacts, and consistent activation timing across repeated flexion–extension cycles. Movement consistency was evaluated by examining cycle-to-cycle elbow angle trajectories and confirming smooth, physiologically plausible forward dynamic behaviour without numerical instability. The dataset selected for simulation did not represent an extreme or optimal case in terms of performance metrics, but rather a technically stable and typical recording suitable for demonstrating the modelling workflow. As no inter-subject statistical comparisons were performed, this approach does not introduce selection bias within the deterministic scenario-based modelling framework.

**Figure 2:**
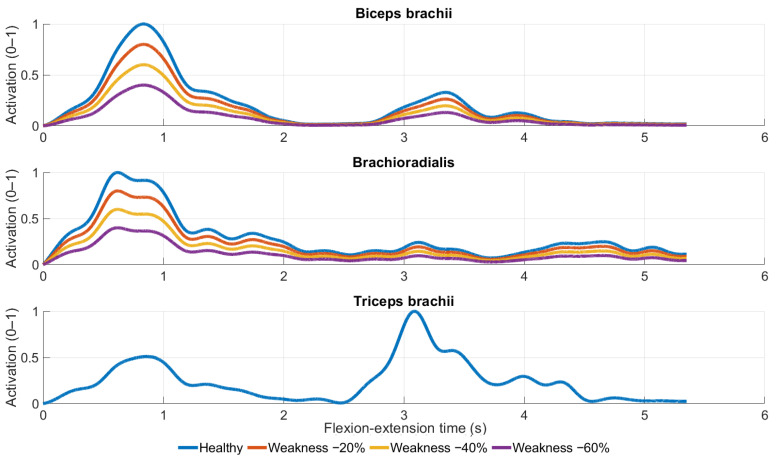
Muscle activation profiles of the elbow flexor and extensor muscles under simulated muscle weakness conditions.

Virtual muscle weakness was simulated by scaling EMG-driven activation of the elbow flexors by −20%, −40%, and −60% relative to the healthy baseline. As intended, scaling of the EMG-driven elbow flexor activation resulted in proportional reductions in activation amplitude without altering activation timing (Figure 2).

Scaling of EMG activation resulted in proportional reductions in peak agonist muscle activation across weakness levels, while activation timing remained unchanged (Figure 2). Antagonist muscle activation showed no relevant differences between conditions.

#### 3.1.1. Elbow Flexion Kinematics Under Virtual Muscle Weakness

**Figure 3:**
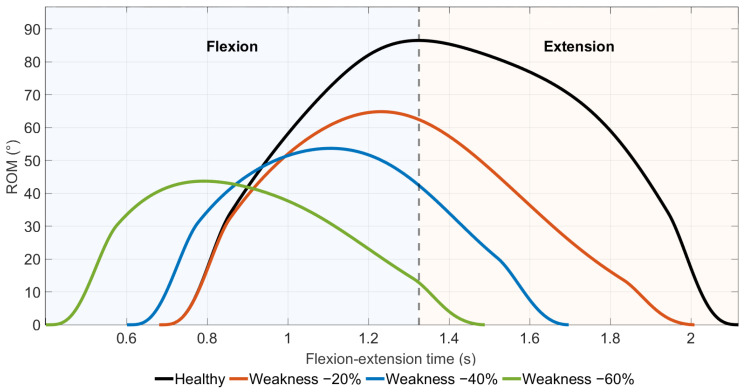
Time-normalized elbow flexion–extension kinematics illustrating the effect of simulated muscle weakness on achievable joint range of motion (ROM).

Virtual muscle weakness resulted in a progressive reduction in elbow flexion amplitude (Figure 3) and an earlier attainment of peak flexion with increasing weakness severity.

Maximum elbow flexion decreased monotonically from 85.2° in healthy conditions to 64.8°, 53.7°, and 43.7° in the −20%, −40%, and −60% weakness scenarios, respectively (Table 3). In parallel, the time required to reach maximum flexion progressively decreased with increasing weakness severity.

The flexion peak time was 5–8% earlier in the −40% and −60% scenarios. In the extension phase, a full return to the starting position was not achieved in the −60% weakness case.

#### 3.1.2. Elbow Flexion Total Moment Under Virtual Muscle Weakness Scenarios

**Figure 4:**
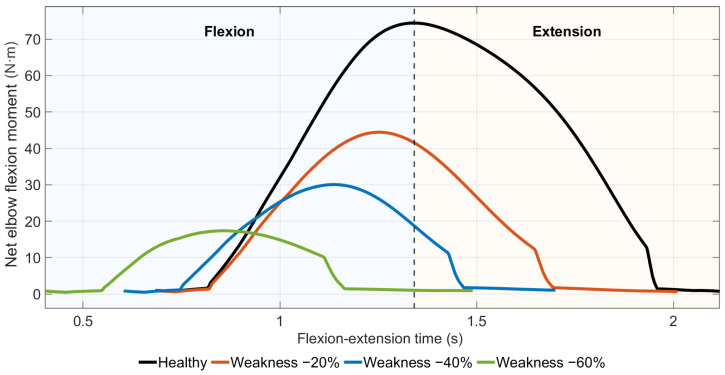
Net elbow flexion moment during EMG-driven forward dynamics under virtual muscle weakness scenarios.

Peak net elbow flexion moment decreased nonlinearly across increasing virtual muscle weakness levels (Figure 4). A 20% reduction in EMG-driven activation led to an approximately 40% reduction in peak joint moment.

Net peak elbow flexion moment decreased from 74.4 Nm in the healthy condition to 44.4 Nm, 30.1 Nm, and 17.4 Nm in the −20%, −40%, and −60% weakness scenarios, respectively.

With increasing weakness severity, the time to peak net elbow flexion moment progressively shifted earlier in the movement cycle, from 1.34 s in the healthy condition to 0.85 s at −60% weakness.

### 3.2. Muscle Activation Patterns Under Increased Antagonist Co-Activation

**Figure 5:**
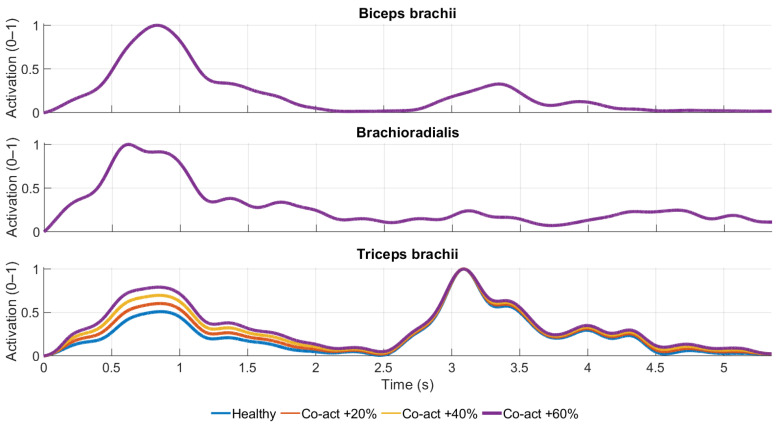
Simulated antagonist co-activation effects on EMG-driven elbow muscle activation patterns.

Agonist activation profiles (biceps brachii and brachioradialis) were identical across all co-activation conditions and overlap in Figure 5, as only triceps brachii activation was selectively increased (+20%, +40%, +60%).

**Figure 6:**
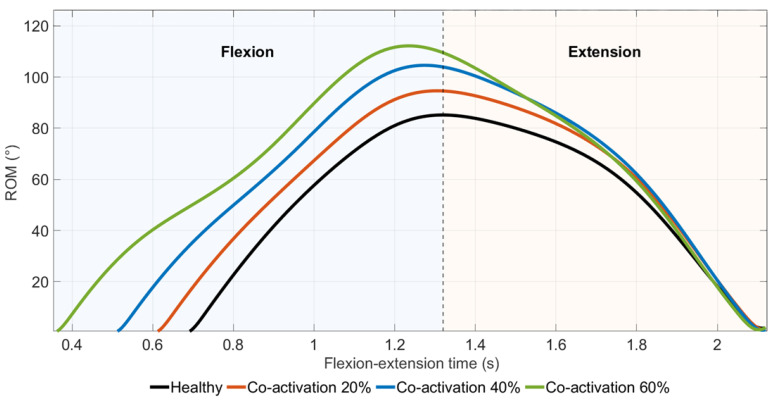
Time-normalized elbow flexion–extension kinematics under virtual antagonist muscle co-activation.

Time-normalized elbow flexion–extension kinematic trajectories obtained under increasing levels of antagonist co-activation are shown in Figure 6. Increasing co-activation levels resulted in progressively larger elbow flexion excursions, with the largest excursion observed in the +60% co-activation condition.

The increased ROM observed under high co-activation conditions may reflect altered joint moment timing and phase-shift interactions between agonist and antagonist muscle groups, rather than pure mechanical stiffening.

Differences between conditions were minimal during movement initiation and termination, whereas the largest deviations occurred during the mid-flexion phase.

#### Elbow Flexion Moment Under Increased Antagonist Co-Activation

**Figure 7:**
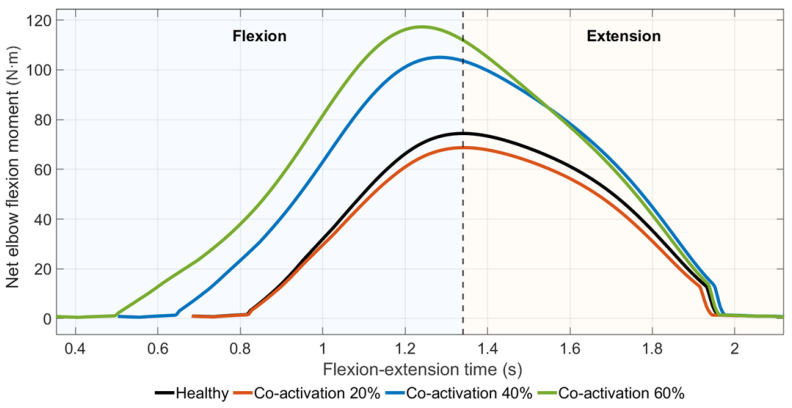
Net elbow flexion moment profiles under increasing antagonist co-activation.

Figure 7 shows net elbow flexion moment profiles obtained from EMG-driven forward dynamics simulations under progressively increased antagonist co-activation (+20%, +40%, and +60%). In contrast to the muscle weakness scenario, increased co-activation resulted in higher peak net elbow flexion moments within the dynamic simulation, reflecting altered phase interactions between agonist and antagonist contributions.

Peak moment values increased from 74.4 Nm in the healthy condition to 104.9 Nm and 117.6 Nm at +40% and +60% co-activation levels, respectively, while a smaller peak moment was observed at the +20% level.

While antagonist co-activation increased cumulative muscle moments, the net joint moment reflected the algebraic sum of agonist and antagonist contributions.

### 3.3. Elbow Flexion Kinematics Under Spasticity Scenarios

**Figure 8:**
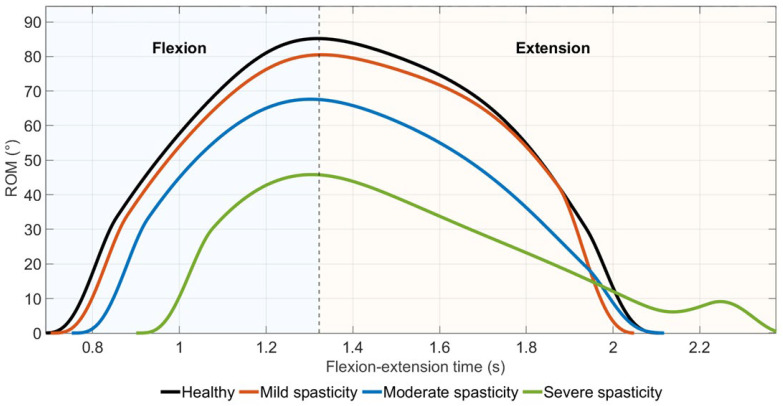
Time-normalized elbow flexion angle across the motion cycle under increasing levels of spasticity-like activation.

Compared to the healthy baseline, increasing spasticity severity resulted in a progressive reduction in peak elbow flexion angle, with an earlier occurrence of peak flexion in the severe spasticity condition (Figure 8). While mild spasticity preserved a movement pattern qualitatively similar to the healthy condition, moderate and severe spasticity led to a markedly constrained range of motion (ROM) and altered trajectory symmetry.

In the severe spasticity scenario, the elbow did not reach mid-range flexion and slowed an early decline in joint angle within the movement cycle.

#### Elbow Flexion Moment Under Spasticity Scenarios 

**Figure 9:**
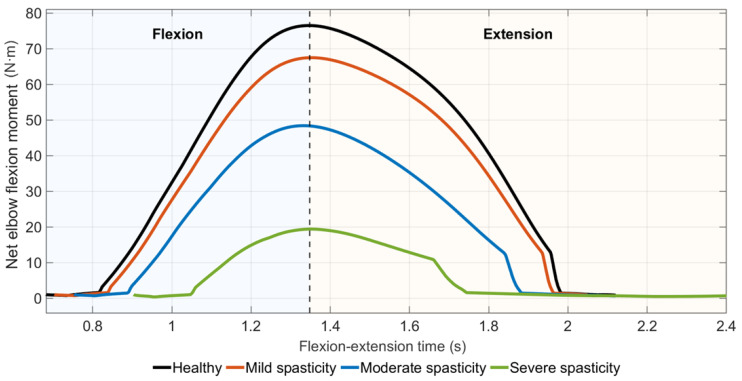
Net elbow flexion moment profiles under increasing levels of spasticity-like activation.

Peak net elbow flexion moment decreased monotonically with increasing spasticity severity (Figure 9). The mild, moderate, and severe spasticity scenarios showed progressively lower peak moment values and shorter durations of elevated flexion moment.

### 3.4. Tremor Related EMG Activation Patterns

**Figure 10:**
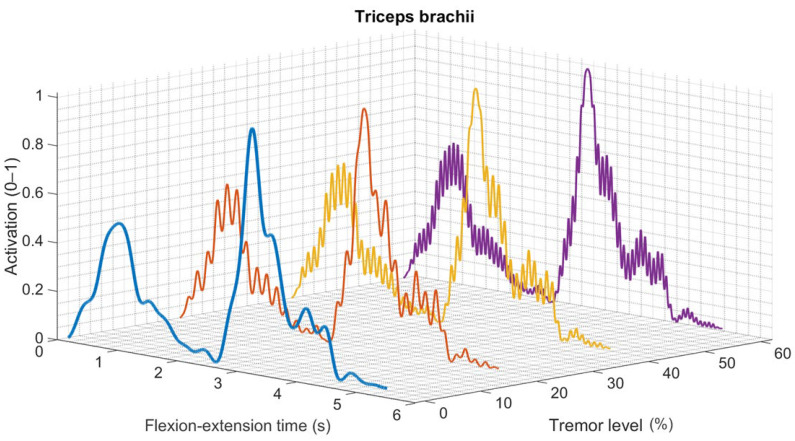
Tremor-related EMG activation patterns across increasing tremor levels.

Tremor-related conditions introduced oscillatory modulation of muscle activation, most clearly visible in the triceps brachii activation profile (Figure 10). Compared with the healthy condition, tremor scenarios introduced high-frequency oscillations into the activation envelope with oscillation amplitude increasing from 20% to 60%.

Oscillatory activity was present throughout the movement cycle and showed larger amplitudes during the mid and late flexion phases. The mean activation level remained similar in all conditions. With increasing tremor intensity, higher-frequency fluctuations became more prominent in the EMG signal.

#### 3.4.1. Elbow Flexion Kinematics Under Tremor Conditions

**Figure 11:**
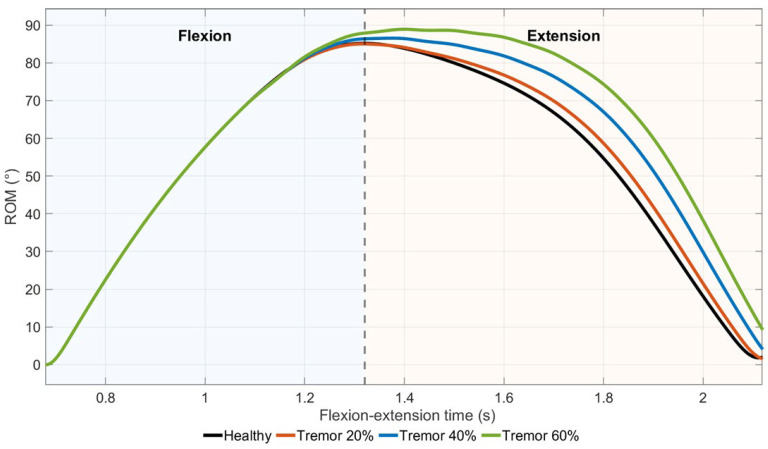
Time-normalized elbow flexion–extension kinematics under increasing tremor levels.

Elbow flexion/extension kinematic profiles under tremor conditions are shown in Figure 11. Time-normalized joint angle trajectories exhibited a consistent unimodal shape across all conditions, with peak elbow flexion occurring at similar phases of the movement cycle.

A higher peak elbow flexion angle was observed at higher tremor intensity levels, with the largest value in the Tremor 60% condition.

#### 3.4.2. Elbow Flexion Moment Characteristics

**Figure 12:**
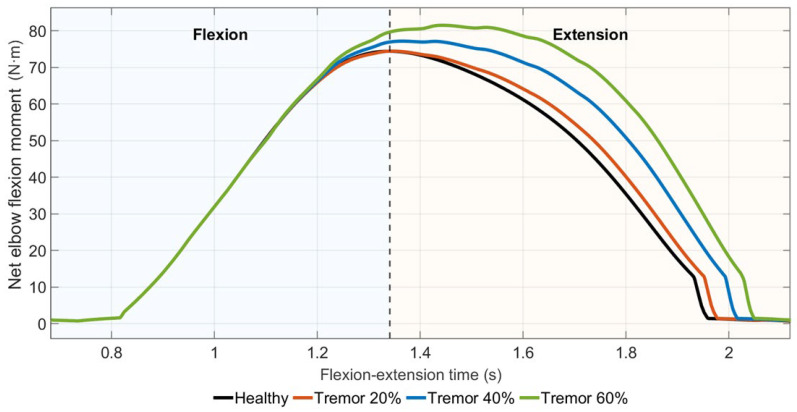
Net elbow flexion moment profiles under increasing tremor levels.

Net elbow flexion moment profiles under tremor conditions are shown in Figure 12. The healthy condition exhibited a smooth net elbow flexion moment profile, whereas tremor scenarios showed higher peak net moments with superimposed oscillatory fluctuations. These oscillatory deviations were superimposed on the net moment envelope and increased with tremor intensity.

Quantitatively, the peak net elbow flexion moment increased progressively from the healthy condition to the Tremor 60% scenario. A shift in the timing of the peak net elbow flexion moment was observed with increasing tremor severity. Tremor conditions were characterized by elevated net elbow flexion moments with superimposed oscillatory fluctuations.

### 3.5. Movement Quality Index (MQI)

**Figure 13:**
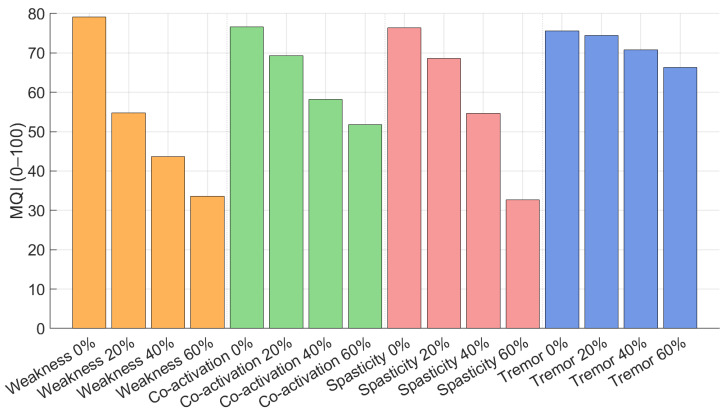
MQI across all clinical scenarios. Colors indicate different impairment mechanisms: muscle weakness (orange), antagonist co-activation (green), spasticity (red), and tremor (blue).

Movement Quality Index (MQI) values across all simulated virtual clinical scenarios are shown in Figure 13.

#### 3.5.1. Muscle Weakness Scenarios

In muscle weakness simulations, MQI decreased with increasing weakness severity. The healthy condition reached an MQI of approximately 79. At −20% weakness, MQI decreased to approximately 55, and further decreased to 44 and 34 at −40% and −60% weakness, respectively.

#### 3.5.2. Co-Activation Scenarios

Increased antagonist co-activation was associated with a moderate decline in MQI. MQI decreased from approximately 76 in the healthy condition to 69, 58, and 52 at low, moderate, and high co-activation. Compared to muscle weakness, the decline was less steep.

#### 3.5.3. Spasticity Scenarios

Spasticity simulations showed one of the largest decreases in MQI. MQI decreased from 76 in the healthy condition to 69, 55, and 33 in mild, moderate, and severe spasticity scenarios, respectively. MQI values in severe spasticity were similar to those observed in severe muscle weakness.

#### 3.5.4. Tremor Scenarios

Tremor scenarios were characterized by a smaller decrease in MQI compared to other conditions. MQI decreased from approximately 75 in the healthy condition to 74, 71, and 66 at tremor intensities of 20%, 40%, and 60%, respectively. The MQI decrease was primarily driven by changes in the stability component.

The numerical values used to calculate the MQI for all simulated scenarios are summarized in Table 4.

## 4. Discussion

This study demonstrates that a subject-specific EMG-driven upper limb musculoskeletal model can reproduce distinct virtual neuromuscular impairment scenarios in a controlled manner. The direct use of EMG signals as control input preserves a transparent relationship between muscle activation and joint mechanics, consistent with early EMG-driven modelling frameworks [19,20]. In the present implementation, personalization was limited to subject-specific EMG input, while musculotendon parameters and model geometry were not individualized.

The increased flexion excursion observed under elevated co-activation conditions likely reflects altered timing and phase interactions between agonist and antagonist activation within dynamic simulation rather than purely increased static joint stiffness.

The implemented activation modifications should not be interpreted as physiological reconstructions of specific neuromuscular disorders, but rather as controlled virtual representations of altered activation patterns designed to investigate their biomechanical consequences.

Although EMG-driven musculoskeletal modelling has been extensively investigated in prior work, particularly in frameworks developed by Buchanan, Lloyd and colleagues, the present study differs conceptually and practically in several key aspects. Classical EMG-driven models primarily estimate muscle forces and joint moments through calibration and optimization procedures that map EMG signals to muscle force outputs. In contrast, the framework proposed here uses experimentally recorded EMG signals directly as prescribed activation inputs in deterministic forward dynamic simulations, without subject-specific force calibration or optimization-based recruitment strategies.

Furthermore, rather than reconstructing muscle forces from recorded movement, the present approach systematically generates controlled parametric activation modifications to simulate and compare distinct virtual activation scenarios within a unified biomechanical context. The introduction of the Movement Quality Index (MQI) enables integrative comparison across scenarios by combining kinematic, kinetic, and stability components into a single research-oriented metric. Thus, the novelty of the framework lies in its structured scenario-based biomechanical differentiation methodology rather than in the use of EMG-driven control itself.

Spasticity scenarios revealed that joint moment generation is more affected than range of motion. In the present simulations, peak joint moment decreased proportionally more than ROM under severe spasticity-like activation modulation. This pattern is mechanically comparable to observations described in the literature [15,21].

The results of co-activation showed that increasing antagonist activity resulted in higher peak net elbow flexion moments within this dynamic simulation framework, likely reflecting altered temporal interactions between agonist and antagonist activation rather than purely increased torque production This observation is consistent with established biomechanical interpretations of antagonist co-activation [22]. However, the present results suggest that such stabilization is achieved at the cost of increased joint moment magnitude and moment variability.

Tremor scenarios showed that with increasing tremor levels, elbow angle trajectories remain close to the healthy state, while joint moments become significantly more oscillatory. This discrepancy between kinematics and kinetics is consistent with literature suggesting that tremors primarily affect force and activation control rather than movement trajectory. In the present simulations, this resulted in oscillatory mechanical behaviour comparable to that described in tremor-related biomechanical studies. [17,18]. The increased moment variability observed in this study supports the inclusion of the stability component of the MQI as a biomechanically informative indicator within the simulation framework.

The framework does not aim to reproduce neurophysiological mechanisms of pathology, nor to model disease-specific EMG signatures. Instead, it operates within a deterministic forward dynamic biomechanical modelling context, where EMG-derived activation signals are treated as prescribed control inputs. Within this clearly defined scope, the modelling approach is internally consistent and fully reproducible. All simulations were performed under identical structural and mechanical conditions, and differences in outcomes arise exclusively from controlled activation perturbations. Therefore, the reported results are reliable with respect to the system-level biomechanical consequences of modified activation inputs within the computational model.

In the present study, equal weighting of kinematic, kinetic, and stability components was adopted to maintain methodological neutrality and to avoid impairment-specific bias in the composite score. However, alternative weighting schemes could emphasize different functional priorities. For example, increasing the relative weight of the stability component would likely enhance differentiation in tremor-like scenarios characterized by elevated moment variability, whereas emphasizing the kinetic component could accentuate distinctions in weakness or co-activation conditions. Conversely, prioritizing kinematic range might reduce sensitivity to subtle alterations in force control. Therefore, while equal weighting was selected to preserve comparability across scenarios, the MQI framework remains adaptable, and future work may explore clinically informed or task-specific weighting strategies to further refine its discriminatory performance.

The deterministic nature of the forward dynamic simulation eliminates biological variability, which is characteristic of real EMG data and neuromuscular disorders. Therefore, the present results should be interpreted as controlled scenario comparisons rather than stochastic representations of biological behaviour.

### Limitations

This study has several important limitations that must be considered when interpreting the results.

An important modelling simplification adopted in this study concerns the representation of muscle activation patterns used to drive the musculoskeletal model. In addition, pathological neuromuscular conditions were not derived from clinical EMG datasets but were represented through controlled parametric modifications of experimentally recorded EMG signals from healthy participants. These simplifications were intentionally introduced to enable a systematic investigation of activation-dependent biomechanical effects within a deterministic simulation framework.

The present study was not designed to estimate inter-individual variability or to perform population-level statistical inference, as all simulations were conducted within a deterministic modelling framework using representative activation inputs.

In the EMG-driven musculoskeletal model muscle forces and joint moments are directly dependent on pre-processed and normalized EMG signals. While this approach allows for a clear and physiologically interpretable relationship between neuromuscular activation and biomechanical outcomes, it also means that the model does not compensate for possible measurement noise, inaccuracies, or EMG-force nonlinearities, which could be partially mitigated by hybrid approaches combining EMG-driven control with optimization-based constraints.

All pathological scenarios (muscle weakness, co-activation, spasticity, and tremor) were simulated synthetically by modifying EMG signals from a healthy individual. Although this approach allows for a systematic and controlled study of the influence of individual neuromuscular factors, it cannot fully reflect complex clinical conditions, which often coexist with structural muscle changes, sensory feedback alterations, and central nervous system adaptations.

The model does not assess changes in muscle-tendon properties that are characteristic of long-term pathological conditions, such as spasticity or chronic paresis. Changes in muscle stiffness, tendon elasticity, and passive moments were not individualized in this work, so biomechanical effects, particularly during extension, may be underestimated.

The study was limited to a single joint (elbow) and a single degree of freedom of movement, so the results obtained cannot be directly generalized to more complex, multi-joint movements of the upper limb, such as reaching or throwing. However, the selected movement allowed for a clear separation and interpretation of the effects of individual neuromuscular mechanisms without additional kinematic interactions.

Extension of the MQI framework to multi-joint tasks would require joint-specific normalization and weighting strategies reflecting task-dependent mechanical contribution. Such expansion represents a future direction for broader biomechanical application.

Despite these limitations, the presented method provides a solid foundation for further studies that could integrate real clinical EMG data, individualized muscle-tendon properties, and hybrid optimization methods.

## 5. Conclusions

This study presented a subject-specific EMG-driven upper limb musculoskeletal model implemented in the OpenSim environment, in which normalized EMG signals were used as direct control input for forward dynamics simulations. This approach preserves a physiologically interpretable relationship between muscle activation and joint-level biomechanics while avoiding reliance on optimization-based muscle recruitment.

Muscle weakness primarily reduced joint moment generation and movement amplitude, antagonist co-activation increased net joint moments with relatively preserved kinematics, spasticity-like activation modulation resulted in pronounced phase-dependent moment attenuation, and tremor predominantly affected joint moment stability while largely preserving kinematic trajectories. These findings indicate that, within the simulation framework, kinematic descriptors alone are insufficient to differentiate activation-modulated scenario mechanisms without complementary kinetic information.

The proposed Movement Quality Index (MQI) enabled an integrated and quantitative comparison of kinematic performance, joint moment magnitude, and stability across scenarios. The MQI consistently reflected scenario-specific changes in movement quality and may serve as a useful comparative metric in simulation-based movement analysis.

Overall, the proposed EMG-driven modelling framework provides a methodological basis for systematic investigation of virtual activation-modulated impairment scenarios and comparative movement analysis. Future work should focus on integrating clinical EMG data, individualized muscle–tendon properties, and multi-joint movements to further evaluate its applicability in rehabilitation-oriented research.

## Figures and Tables

**Table 1:** Comparison of common clinical assessment tools and EMG-driven modelling.

Method	Clinical Use	Measurement	Limitations	Ability to Reflect Functional Muscle Contribution
Goniometry/ROM measurement	Joint range of motion assessment	Static or active joint angles	Measures only joint excursion. No data on muscle activation, timing, coordination or load. Inter/Intratester variability, landmarking errors [1,2].	No insight into which muscles or how they generate movement.
Dynamometry	Strength testing (isometric force)	Maximal (or sub-maximal) voluntary contraction force	Isolated strength, not task-specific, no information on muscle coordination, or functional movement dynamics [3].	Low—only maximal capacity, not real-life function.
Surface EMG	Muscle activation during tasks, neuromuscular assessment	EMG amplitude, onset/offset, timing	Technical issues (crosstalk, variability, dependency on electrode placement), lack of standardized interpretation, limited biomechanical context. Clinical adoption low despite extensive research [4,5]. These limitations highlight the need for modelling-based approaches that explicitly integrate EMG with musculoskeletal mechanics, rather than relying on signal-level interpretation alone.	Medium—shows activation but without force, moment or joint kinematics, so limited functional insight.
EMG-driven musculoskeletal modelling (this study)	Integrative simulation of muscle activation-force-joint kinetics/kinematics	Processed EMG muscle activations, muscle forces, joint moments and kinematics-movement trajectories	Requires signal processing pipeline, expertise, computational modelling, not yet standard clinical tool	Potentially provides quantitative, functional, subject-specific insight into how muscles contribute to movement.

**Table 2:** Barriers limiting clinical use of EMG.

Barrier Category	Description
Educational	Limited formal training in signal processing and EMG interpretation; lack of standardized educational frameworks; communication gap between engineers and clinicians [4].
Technical/Methodological	Signal susceptibility to crosstalk, electrode placement variability, skin–electrode interface effects, normalization inconsistencies, and inter-session variability [5,7].
Interpretational limitations	EMG amplitude does not directly reflect muscle force, joint moments, or mechanical output without biomechanical modelling context [5].
Limited availability of clinically validated integrative tools	Absence of standardized, clinically adopted frameworks that combine EMG with quantitative biomechanical modelling for routine functional assessment [4,5].

**Table 3:** Peak elbow flexion angle and time to peak in the muscle weakness scenarios.

Condition	Peak Flexion, °	Time to Peak, s
Healthy	85.18	1.319
Weakness −20%	64.83	1.233
Weakness −40%	53.67	1.103
Weakness −60%	43.69	0.793

**Table 4:** Numerical components of the Movement Quality Index (MQI) across all simulated clinical scenarios.

	Condition	MQI (0–100)	Kinematic Score	Moment Score	Stability Score	Peak Elbow Moment, Nm	Oscillation Index
Weakness	Healthy	79.14	1.00	1.00	0.165	74.41	0.077
	−20%	54.71	0.77	0.60	0.126	44.43	0.081
	−40%	43.67	0.73	0.40	0.020	30.07	0.091
	−60%	33.59	0.64	0.23	0.000	17.38	0.092
Co-activation	Healthy	76.64	1.00	1.00	0.066	74.41	0.077
	Level 1	69.35	0.93	0.92	0.000	68.72	0.082
	Level 2	58.18	0.86	0.59	0.122	105.02	0.072
	Level 3	51.80	0.83	0.42	0.156	117.27	0.070
Spasticity	Healthy	76.38	1.00	1.00	0.055	76.48	0.075
	Mild	68.56	0.94	0.88	0.000	67.47	0.080
	Moderate	54.66	0.81	0.63	0.008	48.40	0.079
	Severe	32.66	0.54	0.25	0.062	19.41	0.075
Tremor	Healthy	75.54	1.00	1.00	0.022	74.41	0.077
	20%	74.48	0.98	1.00	0.019	74.44	0.077
	40%	70.76	0.92	0.96	0.017	77.17	0.078
	60%	66.30	0.87	0.90	0.000	81.50	0.079

## Data Availability

The raw data supporting the conclusions of this article will be made available by the authors on request.
